# The Prognostic Impact of Protein Expression of E-Cadherin-Catenin Complexes Differs between Rectal and Colon Carcinoma

**DOI:** 10.1155/2010/616023

**Published:** 2010-08-16

**Authors:** Rolf Aamodt, Johan Bondi, Solveig Norheim Andersen, Arne Bakka, Geir Bukholm, Ida R. K. Bukholm

**Affiliations:** ^1^Faculty Division Akershus University Hospital, University of Oslo, 1474 Nordbyhagen, Norway; ^2^Department of Digestive Surgery, Akershus University Hospital, 1478 Loerenskog, Norway; ^3^Department of Pathology, Akershus University Hospital, 1478 Loerenskog, Norway; ^4^Institute of Health and Society, University of Oslo, Forskningsveien 3A, 0373 Oslo, Norway; ^5^Norwegian Unit for Patient Safety, Norwegian Knowledge Centre for the Health Services, Box 7004 St.Olavsplass, 0130 Oslo, Norway; ^6^Department of Health Promotion, Akershus University Hospital, 1478 Loerenskog, Norway

## Abstract

The E-cadherin-catenin complex provides cell-cell adhesion. In order for a carcinoma to metastasize, cancer cells must let go of their hold of neighboring cells in the primary tumor. The presence of components of the E-cadherin-catenin complex in 246 rectal adenocarcinomas was examined by immunohistochemistry and compared to their presence in 219 colon carcinomas. The expression data were correlated to clinical information from the patients' records. There were statistically significant differences in protein expression between the rectal and the colon carcinomas regarding membranous *β*-catenin, *γ*-catenin, p120-catenin, and E-cadherin, as well as nuclear *β*-catenin. In the rectal carcinomas, there was a significant inverse association between the expression of p120-catenin in cell membranes of the primary tumors and the occurrence of local recurrence, while membranous protein expression of *β*-catenin was inversely related to distant metastases.

## 1. Introduction

The E-cadherin-catenin complex provides cell-cell adhesion. It is located at the cell membrane of epithelial cells and consists of a chain of proteins. The outermost element is E-cadherin, also called uvomorulin, which is a transmembrane glycoprotein [1]. E-cadherin protrudes outside the cell membrane and adheres to E-cadherin from neighboring cells through calcium-dependent homophilic interaction. The inner end of the protein chain connects to actin filaments of the cytoskeleton in cytosol [2]. Catenins are proteins in the middle of the chain. They connect actin to E-cadherin [3–8]. A molecule called Eplin binds *α*-catenin to actin [9]. *α*-catenin is connected to either *β*-catenin [10, 11] or *γ*-catenin, also called plakoglobin [3], which in turn are connected to E-cadherin. Distinct from this, *β*-catenin [12] and *γ*-catenin [13] also are translocated to the nucleus and participate in the Wingless pathway.

In the Wingless (=Wnt) pathway, *β*-catenin, that is not bonded, translocates from the cytosol to the nucleus. Here, in a complex with T cell factor (TCF) and Lymphoid enhancer-binding factor (LEF), *β*-catenin controls expression of several genes, among them Snail1 [14]. Snail1 increases the expression of the Forkhead box C2 (FOXC2) gene. So do TGF-beta1, Twist, and Goosecoid. Overexpression of FOXC2 induces epithelial-mesenchymal transition (EMT) [15]. In EMT, an epithelial cancer cell loses its epithelial characteristics, among them adherens junctions, and acquires traits of mesenchymal cells and fibroblasts. This facilitates metastasis. Snail1 represses the transcription of E-cadherin [16, 17]. The reduction in E-cadherin induces a positive feedback loop by its liberation of *β*-catenin that would otherwise be combined with E-cadherin in the E-cadherin-catenin complex by the cell membrane [18]. The liberated *β*-catenin can enter the Wingless pathway, so causing increased expression of Snail1.

p120-catenin stabilizes E-cadherin by controlling E-cadherin turnover at the cell membrane. p120 absence dramatically accelerates E-cadherin degradation [19, 20]. p120 also contributes to supply the cell surface with E-cadherin by recruiting kinesin to cadherin-catenin-containing vesicles [21]. p120 is also supposed to play a role in carcinogenesis independent of E-cadherin. However, the mechanism is still not fully understood [22]. p120 can also be translocated to the nucleus to interact with transcription factor Kaiso [23]. Mild overexpression of Kaiso enhances *β*-catenin signaling, whereas higher levels of Kaiso expression inhibit *β*-catenin signaling [24].

Adherens junctions are cellular structures found near the apical surface of polarized epithelial cells. They provide cell-cell adhesion. The E-cadherin-catenin complex is typically found in adherens junctions [25, 26], but the E-cadherin-catenin complex also appears outside adherens junctions [4, 26, 27].

In order for a carcinoma to metastasize, cancer cells must detach from neighboring cells in the primary tumor. This process requires a malfunction of the E-cadherin-catenin complex. Several studies have demonstrated reduced expression of E-cadherin [28–35] and catenins [32–34, 36–43] in a number of carcinomas. All these studies indicate that E-cadherin/catenin-mediated cell adhesion is crucial in development and progression of human carcinomas [44], and E-cadherin acts as an invasion and metastasis suppressor molecule in cancer [26, 45–47]. p120 loss appears to be an early event in tumor progression [48].

A simultaneous investigation of the protein expression of both E-cadherin and all the catenins in the E-cadherin-catenin complex in only rectal carcinomas has so far never been performed. Most of the previous studies have included both rectal and colon carcinomas. Since there are clinical differences in prognosis and outcome between patients operated for rectal and colon carcinomas, as well as reports about tumor biological differences between these two tumor types [49], we aimed in the present study at evaluating the expression of proteins known to play a pivotal role in the metastatic process, in rectal adenocarcinomas solely. We also wanted to explore whether there may be differences in expression patterns of these proteins between rectal and colon adenocarcinomas.

## 2. Materials and Methods

### 2.1. Patient Materials

Available paraffin-embedded tumor samples from a consecutive series of 274 rectal adenocarcinomas removed surgically at Akershus University Hospital in the years 1992–2000 were scrutinized for inclusion into the survey. These surgical treatments were all primary operations. Tumors at a level of 15 centimeters (5.9 inches) or less from the anal verge (i.e., the outer border of the anus) were included (246 patients), in accordance with the somewhat arbitrary range of 15 to 18 centimeters commonly used to define the border between rectum and colon. The most restrictive border was chosen in order to avoid unintentional inclusion of sigmoid tumors. The clinico-histopathological characteristics of the patients are shown in Table 1.

Out of 246 patients, 25 experienced a local recurrence of their rectal cancer. Among those included in the statistical analyses, mean time from primary operation to local recurrence was 2.4 years. Minimum time was six months, maximum 6.0 years. For patients with no local recurrence, mean observation time was 6.7 years (range 4 days–14.5 years). Out of 246 patients, 49 developed distant metastases from their rectal cancer. Among those included in the statistical analyses, mean time from primary operation to distant metastases was 2.3 years. Minimum time was two months, maximum 12.9 years. For patients without distant metastases, mean observation time was 7.2 years. Minimum observation time was four days, maximum 14.4 years.

This material of rectal carcinomas was compared to a material examined by Bondi et al. of 219 colon carcinomas operated on at Akershus University Hospital during the years 1988, 1990, and 1997–2000 [28, 50].

### 2.2. Immunohistochemistry

Serial sections (3-4 micrometers) from formalin fixed, paraffin wax embedded archive tumor tissue were applied to coated slides before immunohistochemical staining, where different methods were used.

For *β*-catenin, sections were dried at 50–60°C overnight, deparaffinized and rehydrated before antigen retrieval was performed using microwave technique (20 minutes at 100°C). Staining was done in Dako Autostainer (Dako Corporation, Carpinteria, CA).

Sections for *α*-catenin, p120-catenin, and E-cadherin were dried overnight at 50–60°C before pretreatment with Dako PT link (20 minutes in 98°C), and staining in Dako Autostainer (Dako Corporation, Carpinteria, CA), using Dako's EnVision Flex-system, with mouse-linker in p120-catenin and E-cadherin.

For *γ*-catenin, sections were fixed in incubator 30–40 minutes at 56°C, and overnight at 37°C. The immunostaining was performed in Ventana ES Benchmark automated slide stainer (Ventana Medical Systems, Tucson, AZ), antibody diluent (251-018). The details of each antibody used are shown in Table 2.

The antibodies were visualized for light microscopy with Envision Plus-System and diaminobenzidine (DAB), and with Detection Kit Ventana iViewTM DAB, respectively. Counter-staining was done with Hagen's haematoxylin for visualization of tissue structures. Positive control was a test block with normal colon mucosa and multiple colonic adenocarcinomas with diverse differentiation.

The percentage of positive cell membranes was counted semiquantitatively by applying four defined grades of immunopositivity. When 60% or more of the cancer cells were stained, we scored the tumor as grade three. Staining of 30%–59% of the cells was classified as grade two. When 5%–29% of the cells were stained, the score was grade one. Positivity in less than five percent of cells qualified for grade zero. The protein expression was regarded positive regardless of whether the expression was mainly at the apical portion of the cell [51, 52] or if it appeared to be relatively evenly distributed around the cell membrane.

Scoring of nuclear staining of *β*-catenin was done in the same way as for the membranes, except for that the border between grade zero and grade one was set to zero percent of the nuclei being stained. The reason for this is that normally, there should be no staining of nuclear *β*-catenin at all. Only clearly nuclear staining was recorded as positive.

We intended to compare our results from rectal cancers to results from a similar examination of colon cancers previously performed. Therefore, our method for quantifying proteins in question had to be the same, with the same cut-offs between the scored grades. Originally, the choice of four levels of staining were applied as a compromise between the wish of detailed measurements and the wish of not giving the impression of being more accurate than manual human judgment is in real life. One intention with the system was that alterations in protein staining in a cancer should be larger than alterations naturally occurring in normal mucosal tissue before they were recorded as pathological. For most slides, we were able to check that this intention was followed, because almost all slides contained normal adjacent mucosa in addition to the cancer. The normal mucosa served as an internal control. In other, earlier, similar investigations, varying cut-offs have been used, and no unified, generally applied standard seems to exist for this. So, in addition to the above mentioned, the original cut-offs were somewhat arbitrarily chosen.

At least 100, usually more than 1000 cells, were examined in each slide. All slides were primarily judged by RAA. In addition, judgment of slides was done by AB, IRKB, JB, or SNA, depending on the type of slide in question. When necessary, consensus was achieved by simultaneous examination by two examiners in a double microscope.

### 2.3. Statistics

Statistical analyses were performed by SPSS version 16.0 running on Windows XP. Binary logistic regression analysis and Cox regression analysis were performed. We made test plots for proportional hazards for the Cox analyses and found them satisfactory. An alpha level of statistical significance of *P* < .05 was chosen.

In all statistical analyses, we included patient gender, tumor differentiation grade, Dukes' tumor stage, and patient age at surgery as well as the five membrane proteins *α*-catenin, *β*-catenin, *γ*-catenin, p120-catenin, and E-cadherin, and also nuclear *β*-catenin, into the same multivariate analysis.

## 3. Results

**Figure 1:**
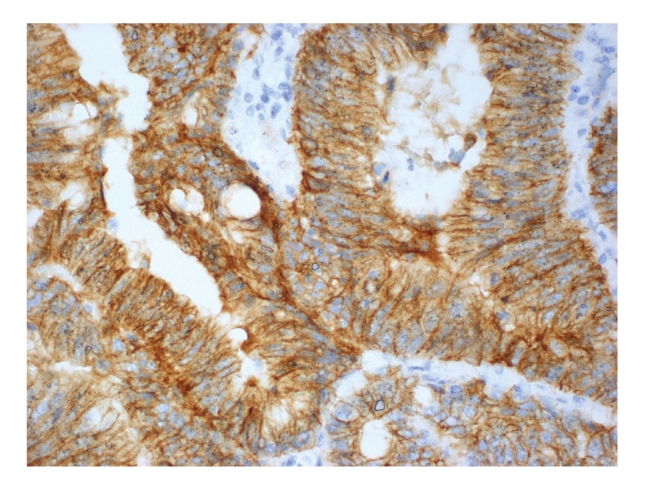
Representative example of immunopositivity grade 3 for *α*-catenin (original magnification x400).

**Figure 2:**
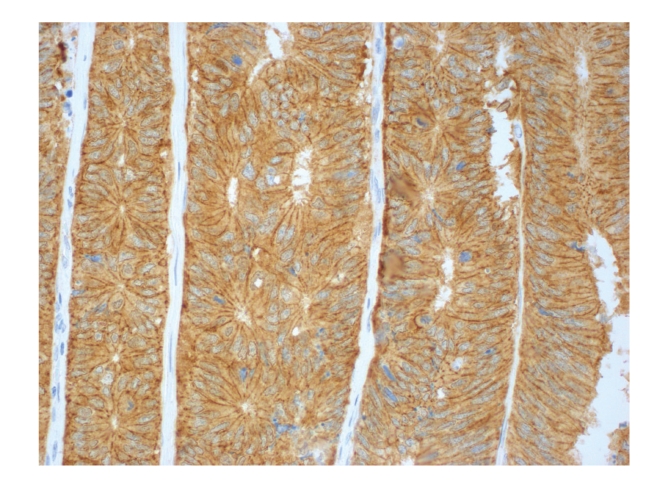
Representative example of immunopositivity grade 3 for *β*-catenin (original magnification x400).

**Figure 3:**
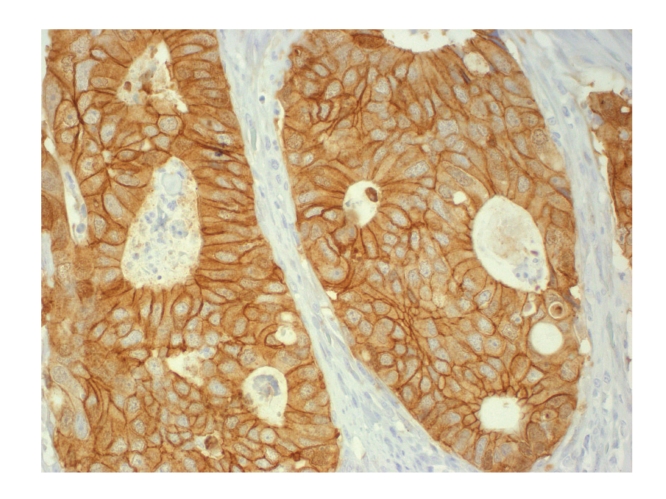
Representative example of immunopositivity grade 3 for *γ*-catenin (original magnification x400).

**Figure 4:**
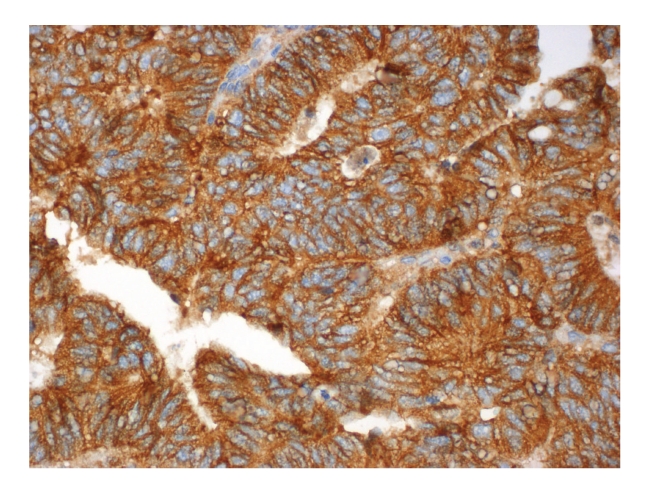
Representative example of immunopositivity grade 3 for p120-catenin (original magnification x400).

**Figure 5:**
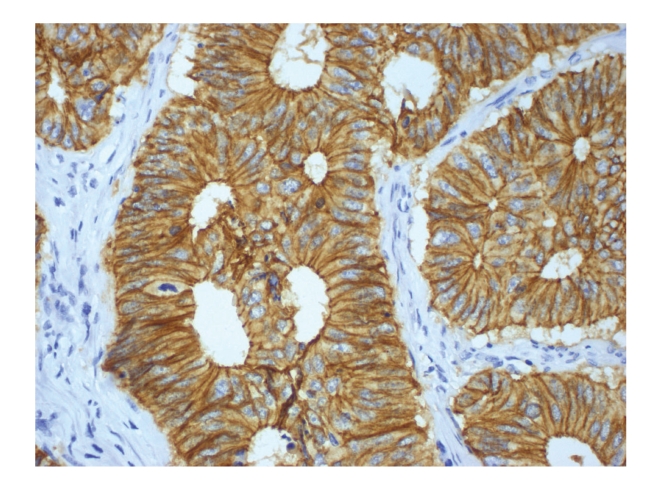
Representative example of immunopositivity grade 3 for E-cadherin (original magnification x400).

Figures 1, 2, 3, 4, and 5 show representative examples of immunostaining. 

The immunohistochemical scores for the membrane proteins as well as nuclear *β*-catenin in the rectal and the colon adenocarcinomas are shown in Table 3. There were significant differences in protein expression between the rectal and the colon cancers regarding membranous staining of *β*-catenin, *γ*-catenin, p120-catenin and E-cadherin, and regarding nuclear staining of *β*-catenin (Table 4). That is, all examined proteins except *α*-catenin showed significant differences.

In the rectal carcinomas, there was a significant inverse relationship between the expression of p120-catenin in cell membranes of the primary tumors and the occurrence of local recurrence (Cox analysis, *P* = .030, HR = 0.492, 95% CI for HR [0.260; 0.932]). There was also a significant inverse relationship between the expression of *β*-catenin in cell membranes of the primary tumors of the rectal cancers and the occurrence of metastases (Cox analysis, *P* = .003, HR = 0.579, 95% CI for HR [0.404; 0.829]). 

## 4. Discussion

Results from the present study indicate differences between rectal and colon cancers in expression patterns of adhesion proteins. The present study also demonstrates, for the first time, that reduced protein expression of p120-catenin in cell membranes of primary rectal carcinomas is associated with more often local recurrence. Reduced protein expression of *β*-catenin in cell membranes of primary rectal carcinomas more often gave distant metastases.

To our knowledge, no previous study exists on p120-catenin in a material solely of rectal cancers. Results regarding p120-catenin in colon and colorectal cancers diverge. In a study exclusively on colon cancers, there was no relationship between the expression of p120-catenin and metastasis or survival [28]. Colorectal cancers, that is, a mixture of colon and rectal cancers, with loss of p120-catenin expression have been found to be more likely metastatic to lymph nodes and distant organs and result in poor survival [53]. An interpretation of these results and the results of the present study could be that reduction of p120-catenin expression is associated with adverse clinical effect only in rectal cancers.

Fernebro et al. found that reduced membranous staining of *β*-catenin in rectal cancer correlated significantly to development of metastases, but only in univariate analysis [54]. This is in accordance with our findings, but we found this relation to be significant in multivariate analysis, where also possible confounders were included in the statistical analysis. Protein expression of *β*-catenin in cell membranes did not associate with patient prognosis in colon cancers [28]. A study of colorectal cancers, that is, a mixture of rectal and colon cancers, showed that loss of membranous expression of *β*-catenin was associated with short survival and death from metastatic colorectal cancer, but only in univariate analysis [33].

Results from the present study indicate a different role of membranous *β*-catenin expression in rectal and colon carcinomas. The same seems to be the case for E-cadherin protein expression. We observed no correlation between protein expression of E-cadherin in rectal tumor tissue and patient prognosis, while several studies have demonstrated a significant association between reduced expression of E-cadherin and impaired patient prognosis in patients operated for colon adenocarcinomas as well as other tumor types [28, 55–59]. The reason for this difference regarding the prognostic value of E-cadherin between rectal and colon adenocarcinomas is difficult to explain, but may be the result of different tumor biology within these two entities.

E-cadherin is one of the key proteins in epithelial-mesenchymal transition (EMT), a process crucial for the metastatic process. The present study indicates that the EMT process may be different in rectal and colon adenocarcinomas within the same tumor stage.

Results from the present study strongly indicate that rectal and colon adenocarcinomas should be separated before analyzing the prognostic value of proteins involved in the adhesion complexes of the cells.

## 5. Conclusions

The present research is one of the first studies demonstrating tumor biological differences with significance on protein level between rectal and colon adenocarcinomas, regarding the metastatic process. These differences in tumor biology may explain some of the differences in clinical behavior of these two tumor types, but such differences are so far not taken into account when planning the treatment options. The observation that p120-catenin may be related to decreased risk of experiencing local relapse may help stratifying patients, together with other modalities, in risk groups for relapse. 

## Figures and Tables

**Table 1:** Rectal and colon cancers' characteristics no. (%).

*Rectal Cancers*				
Gender	Males: 150 (61)	Females: 96 (39)		
Age at operation	Lowest: 16	Mean: 66	Highest: 90	
Dukes' stage	A: 45 (18)	B: 100 (41)	C: 69 (28)	D: 30 (12)
T stage	T1: 8 (3)	T2: 46 (19)	T3: 187 (76)	T4: 5 (2)
N stage	N0: 154 (63)	N1: 63 (26)	N2: 29 (12)	
M stage	M0: 214 (87)	M1: 30 (12)		
Tumor differentiation	Poor: 7 (3)	Moderate: 235 (96)	High: 2 (1)	

*Colon Cancers*				
Gender	Males: 105 (48)	Females: 114 (52)		
Age at operation	Lowest: 40	Mean: 70	Highest: 93	
Dukes' stage	A: 10 (5)	B: 105 (48)	C: 57 (26)	D: 47 (22)
T stage	T1: 4 (2)	T2: 27 (12)	T3: 173 (79)	T4: 14 (6)
N stage	N0: 137 (63)	N1: 65 (30)	N2: 15 (7)	
M stage	M0: 169 (77)	M1: 47 (22)		
Tumor differentiation	Poor: 23 (11)	Moderate: 184 (84)	High: 11 (5)	

**Table 2:** Primary antibodies used for immunohistochemistry.

Antibody	Retrieval method	Dilution	Incubat time	Host species	Clone	Vendor
*α*-catenin	Dako TRS high pH	1 : 150	30 minutes	Mouse, monoclonal	25B1	BD Biosciences Europe*
*β*-catenin	Tris/EDTA pH 9	1 : 300	30 minutes	Mouse, monoclonal	17C2	Novocastra^†^
*γ*-catenin	Tris/EDTA	1 : 25	30 minutes	Mouse, monoclonal	11B6	Novocastra^†^
p120-catenin	Dako TRS high pH	1 : 75	30 minutes	Mouse, monoclonal	15D2	Santa Cruz Biotechnology^‡^
E-cadherin	Dako TRS high pH	1 : 100	30 minutes	Mouse, monoclonal	NCH-384	Dako**

*BD Bio Sciences Europe, Norge@Europe.bd.com.

^†^Novocastra, Newcastle, UK.

^‡^Santa Cruz Biotechnology, Inc., Europe@scbt.com.

**Dako, N-1086 Oslo, Norway.

**Table 3:** Immunohistochemical scores in the rectal and colon adenocarcinomas no. (%).

Protein	Immunohistochemical score				Total
	0	1	2	3	
*Rectal Cancers *					
*α*-catenin	0 (0)	8 (3)	30 (13)	195 (84)	233 (100)
*β*-catenin membranous	4 (2)	10 (4)	8 (4)	204 (90)	226 (100)
*β*-catenin nuclear	12 (5)	130 (55)	47 (20)	46 (20)	235 (100)
*γ*-catenin	1 (0)	6 (3)	5 (2)	221 (95)	233 (100)
p120-catenin	1 (0)	4 (2)	21 (9)	209 (89)	235 (100)
E-cadherin	0 (0)	1 (0)	4 (2)	220 (98)	225 (100)

*Colon Cancers*					
*α*-catenin	56 (28)	35 (17)	65 (32)	46 (23)	202 (100)
*β*-catenin membranous	65 (32)	52 (25)	46 (22)	43 (21)	206 (100)
*β*-catenin nuclear	126 (78)	34 (21)	2 (1)	0 (0)	162 (100)
*γ*-catenin	115 (56)	41 (20)	31 (15)	18 (9)	205 (100)
p120-catenin	105 (54)	53 (27)	18 (9)	19 (10)	195 (100)
E-cadherin	90 (44)	59 (29)	30 (14)	26 (13)	205 (100)

**Table 4:** Differences in expression of membrane proteins and nuclear *β*-catenin between rectal and colon adenocarcinomas. Binary logistic regression.

Protein	Highest in	*P*	OR	95% CI for OR	
*β*-catenin membranous	Rectum	.005	11.999	2.121	67.872
*γ*-catenin	Rectum	.023	3.141	1.167	8.453
p120-catenin	Rectum	.002	7.045	2.009	24.706
E-cadherin	Rectum	0.004	12.996	2.319	72.824
*α*-catenin	Non-significant	.348	1.972	0.478	8.137
*β*-catenin nuclear	Rectum	.004	83.070	4.155	1660.942
